# Human Brain Physiology Investigated in the Disorder of Consciousness

**DOI:** 10.3389/fneur.2014.00211

**Published:** 2014-10-17

**Authors:** Walter G. Sannita

**Affiliations:** ^1^Department of Neuroscience, Ophthalmology and Genetics, University of Genova, Genova, Italy; ^2^Institute S. Anna – Research in Advanced Neurorehabilitation (RAN), Crotone, Italy; ^3^Department of Psychiatry, State University of New York at Stony Brook, Stony Brook, NY, USA

**Keywords:** brain, physiology, disorder of consciousness, neuroimaging, research

Neuroimaging research has shown that subjects in vegetative state (or unresponsive wakefulness syndrome; VS/UWS) may retain high-level aspects of brain activity across sensory modalities, language and learning dynamics, emotions, or pain. Brain responses vary in complexity, from local activation of primary sensory cortices, to the involvement of associative areas, to activation of cortical–subcortical networks to either mental imagery tasks or distinction of ambiguous/non-ambiguous words or figures (1–3).

These observations document the capability of the severely damaged brain to express surviving modular functions despite impaired corticocortical and cortico-subcortical connectivity and are understood as indicative of retained, covert cognition, or consciousness as opposed to interpretations that markers of neural activity not necessarily are surrogates for conscious activity (1–4). The controversy challenges our definitions of phenomenal consciousness, and blurs the extent to which the classical binary conscious/unconscious separation may serve. The implications can entangle the current diagnostic criteria as well as medical care, legal or popular perception of bioethical issues, allocation of human resources and logistics, and healthcare policies for VS/UWS. The issue nevertheless is mainly scientific and remains unsolved (2, 3).

The task- or stimulus-related brain activation in fMRI studies may or may not be indicative of residual, covert consciousness in VS/UWS (2), but neuroimaging research on the disorder of consciousness is certainly seminal. In particular, it should be emphasized how the surviving brain structures and networks identified in VS/UWS compare as to anatomy and modes of activation to those observed in healthy subjects under comparable stimulus/task conditions. This similarity indicates that brain activation is not at random. It also compels the evidence that the brain can operate in sensory data processing or motor action with limited or null control from conscious processes: where and how, in the brain, sensory information processing becomes conscious perception or intentional planning becomes movement remains undocumented (5). In VS/UWS, interference on bottom-up processes from top-down controlling brain systems is peculiarly negligible due to the selective disruption of the top-down projections from high-order associative cortices (6); modulation by attentional processes, spontaneous fluctuations in the brain functional state (e.g., in vigilance), and interaction with the environment are conceivably also irrelevant. In a reversed perspective, the findings of neuroimaging research (and the experimental approach itself) would thus provide unique information about unconscious brain mechanisms or functions that in VS/UWS have been investigated in experimental conditions that happen to be ideally controlled. This rationale should be discussed and submitted to a strict logical interpretation in view of application in research.

## Conflict of Interest Statement

The author declares that the research was conducted in the absence of any commercial or financial relationships that could be construed as a potential conflict of interest.
